# Immunotherapy: Newer Therapeutic Armamentarium against Cancer Stem Cells

**DOI:** 10.1155/2020/3963561

**Published:** 2020-03-09

**Authors:** Saurabh Pratap Singh, Richa Singh, Om Prakash Gupta, Shalini Gupta, Madan Lal Brahma Bhatt

**Affiliations:** ^1^Department of Oral Pathology & Microbiology, King George's Medical University, Lucknow, India; ^2^Centre for Integrated and Translational Research, CSIR-Indian Institute of Toxicology Research, Lucknow, India; ^3^Depatment of Surgery, Prasad Institute of Medical Sciences, Lucknow, India; ^4^Vice Chancellor, King George's Medical University, Lucknow, India

## Abstract

Mounting evidence from the literature suggests the existence of a subpopulation of cancer stem cells (CSCs) in almost all types of human cancers. These CSCs possessing a self-renewal capacity inhabit primary tumors and are more defiant to standard antimitotic and molecularly targeted therapies which are used for eliminating actively proliferating and differentiated cancer cells. Clinical relevance of CSCs emerges from the fact that they are the root cause of therapy resistance, relapse, and metastasis. Earlier, surgery, chemotherapy, and radiotherapy were established as cancer treatment modalities, but recently, immunotherapy is also gaining importance in the management of various cancer patients, mostly those of the advanced stage. This review abridges potential off-target effects of inhibiting CSC self-renewal pathways on immune cells and some recent immunological studies specifically targeting CSCs on the basis of their antigen expression profile, even though molecular markers or antigens that have been described till date as expressed by cancer stem cells are not specifically expressed by these cells which is a major limitation to target CSCs. We propose that owing to CSC stemness property to mediate immunotherapy response, we can apply a combination therapy approach by targeting CSCs and tumor microenvironment (TME) along with conventional treatment strategies as an effective means to eradicate cancer cells.

## 1. Introduction

Cancer is a diverse heterogeneous disease which is characterized by phenotypically and functionally discrete subsets of cells. Data amassed from the literature suggests the presence of a small population of cancer cells with stemlike properties in a wide continuum of human cancers. Characterized by self-renewal and differentiation, these cells have been termed as cancer stem cells or tumor-initiating cells, and we have used CSCs to denote these cells throughout the review. CSCs are biologically similar to normal stem cells (SCs) [1, 2]. CSCs are characterized functionally by the intrinsic ability to initiate and long-term repopulate tumors with a recapitulation of the lineage/cellular heterogeneity seen in parental tumors [3]. Accumulated evidences advocate that from the time when CSCs were initially identified in human acute myeloid leukemia (AML), they have been isolated from many divergent malignancies, including cancers of the breast, prostate, colon, brain, pancreas, lung, liver, bladder, and ovary [4–8].

CSCs also appear to have resistance to anticancer therapies leading to relapse. This deleterious feature of CSC causes a dire impact on cancer management and hence makes CSCs promising targets for elimination. There are a few ongoing trials that involve immunotherapy strategies against CSCs [9]. However, in order to design newer therapeutic approaches, we need a clearer understanding of the biology of these cells. The present review aims to determine the feasibility of immune targeting CSCs in solid tumors and also highlights that some of the biological targetings of CSCs may be ambivalent by also affecting immune responses.

## 2. Role of Developmental Signalling Pathways in the Regulation of CSCs

Tumorigenesis bears resemblance to abnormal organogenesis. CSCs exhibit three cell-intrinsic fundamental properties: self-renewal, quiescence, and differentiation. Therefore, any genetic or epigenetic program that can regulate one or more of these three properties could theoretically have an impact on CSC biology [3]. CSC phenotypes change due to altered genetics via various mechanisms. CSC biology is majorly governed by developmental pathways, stem cell factors, cell cycle regulation and apoptosis, epithelial-mesenchymal transition (EMT), and epigenetics apart from physiological metabolism. Owing to complex interactions and overlap between mechanistic programs driving CSC regulation, defining five mechanisms is rather arbitrary, as they could all ultimately converge on transcriptional regulation driven by myriad transcription factors [1].

An array of signalling pathways, namely, Myc, Notch, Hedgehog (Hh), Wnt, FGF/FGFR, EGF/EGFR, NF-*κ*B, MAPK, PTEN/PI3K, HER2, and JAK/STAT operative in normal SCs during development and homeostasis are frequently found to be deregulated in CSCs [10–14]. Developmental pathways such as Notch, Wnt/*β*-catenin, and Hedgehog participate instrumentally in cancers and are frequently altered and are implicated in CSCs regulation [15–18].

Notch ligands, residing in plasma membrane of neighbouring cells, are transactivated, eliciting the transcription of Notch target genes, such as c-myc, p21 and p27, PI3K, the hairy and enhancer of split- (HES-) related family, protein kinase B (AKT), peroxisome proliferator-activated receptor (PPAR), NF-*κ*B, and cyclin D1. Depending on the particular signalling milieu, these downstream targets get activated and in turn regulate cell fate leading to differentiation, cell-cycle progression, and survival. Notch may decelerate differentiation and endorse cell survival in stem-like cells [19, 20]. There are numerous ongoing Phase I and II clinical trials in cancer with a range of targets and mechanisms investigating the efficacy of Notch targeting, singly or in amalgamation with other therapeutic strategies [19]. Notch signalling has, nevertheless, also been correlated to peripheral T-cell maturation into effector cells, such as developing cytotoxic T-cell function, or cytokine production [21]. Similarly, T-cell activity has been weakened by Notch-inhibition using *γ*-secretase inhibitor [22].

Two signalling pathways have been recognized which are the *β*-catenin-dependent pathway that is involved in cell fate determination (canonical), and the *β*-catenin independent pathway that is involved in cell polarity and movement (noncanonical). Commencement of Wnt signalling is fostered by the release of soluble ligands by the cells in the near vicinity. The gain of function, regulation by methylation, and histone modification have all been implicated in carcinogenesis due to this pathway [17, 23, 24]. The focus of treatment research in cancer is to inhibit the adverse pathway in CSCs [25]. However, the Wnt/*β*-catenin pathway is a double-edged sword as it functions as a major player in the regulation of T-cell development and activation, Wnt/*β*-catenin signalling is crucial for CD8^+^ memory T-cell development, and on the other hand, the efficacy of immunotherapy responses is improved by agonists [26, 27].

The Hedgehog (Hh) signalling pathway plays a key role in tissue wear and tear and homeostasis and epithelial to mesenchymal transition (EMT) in normal tissues. Belligerent manifestations like cancer may be prompted by tumorigenesis via aberrant Hh signalling defined by the overexpression or loss of function of its ligands or receptor and dysregulation of transcription factors. Hh signalling can be triggered by an array of factors in the tumor microenvironment, such as transforming growth factor- (TGF-) *β*, tumor necrosis factor- (TNF-) *α*, and interleukin (IL)-6. Attenuation of Hh signalling is also attracting focus of researchers worldwide and undergoing intense exploration for cancer treatment [28]. Hh signalling is relevant in immune cell development and function [29–32]. Also, Hh inhibitors may deliver additional benefits as they are also involved in myeloid-derived suppressor cell (MDSC) function. Singly targeting any pathway may not yield a physiologically relevant level of inhibition as there is a considerable overlap between these pathways. Since it is responsible for normal tissue homeostasis and development, including immune cell behaviour and peripheral effector function, targeting them is a challenging job [33].

## 3. Identification and Isolation of CSCs

### 3.1. Identification Based on Immunophenotypic Marker

Coherent immune targeting of CSCs depends on the categorization of (i) stem cell-like CSC markers used for its isolation and (ii) Antigens expressed on CSCs which are not preferably express on non-CSC/normal cells. The SC biology of the tissues from which tumor originates forms the basis of identification of CSC markers since the lineage relationship between CSCs and tissue SCs remains vague in most tumor systems. For detection and segregation of alleged CSC populations, not only are functional assays like ALDEFLUOR, mammosphere, and neurosphere employed, but also side population (SP) and cell surface marker coupled with a fluorescence-activated cell sorter (FACS) are put to use. The immunophenotypic markers for potential CSCs have been reported in many human malignancies [1, 3, 6–8] (Table 1). A range of CSC markers (CD44, CD133, and HER2) represent promising targets for CSC immunotherapy, such as antibody therapy. However, probable safety issues should be addressed when utilizing these markers as CSCs since normal SCs share similar phenotypic marker profiles. Also, a limited number of CSC markers in solid malignancies are currently accessible and their biological function is less categorized.

It is a well-established notion that CSCs in different tumor types demonstrate distinctive transcriptomic profiles and thereby express discrete antigens. They express a continuum of tumor-associated antigens (TAAs) that have the capacity to be identified by the immune system of the host [3]. TAAs may be categorically illustrated in CSCs as four subgroups [3, 8, 31], namely, (i) nominal expression by normal tissues but constitutively highly expressed by tumors as part of being their malignant phenotype like EGFR and surviving, (ii) differentiation antigens that are tissue-specific and expressed by both benign and cancer cells, (iii) CT cancer/testis antigens inadvertently activated in tumors like MAGE-A3 and MAGE-A4 that are normally confined to placenta and testicular germ cells, and (iv) neoantigens in cancer genomes derived from mutations leading to new epitopes identifiable by immune system like MUM-1 and CDK4 in melanoma. Innumerable lineage-specific markers are categorised as differentiation antigens, namely, PSA in PCa and MART-1 in melanoma. CSC markers can henceforth be thought to act as overexpressed antigens although not all are expressed minimally in normal cells. CT antigens and neoantigens have been considered best for CSC-targeting immunotherapy although lineage differentiation antigens are expressed in low amounts in CSCs [3, 8, 31].

### 3.2. Identification Based on Metabolism

There is a metabolism-based identification which is based on the activity of certain enzymes such as aldehyde dehydrogenase (ALDH) and mitochondrial glycolytic activity.

The ALDH family is encompassed by a gamut of 19 genes (in humans) that articulate enzymes for catalysis of aldehyde oxidation. Metabolism of vitamins, amino acids, and lipids requires aldehyde oxidation [34]. Elevated ALDH activity is linked with both normal SCs and CSCs. These enzymes also exert a shielding detoxifying effect by catabolizing aldehydes derived from pharmacological substrates. Identification of CSCs in the liver, breast, colon, and head and neck is done measuring ALDH activity employing flow cytometry-based ALDEFLUOR assay. This method is more instantaneous and is the direct readout of a fluorescent signal, based on enzyme activity as compared to other phenotypical detection methods. This method is less prone to discrepancies encountered by antibody-based staining, namely, epitope downregulation or masking, or expression of splice variants. The ALDEFLUOR assay uses N,N-diethylaminobenzaldehyde (DEAB) to measure the activity of the ALDH1 family for which it acts as a specific inhibitor [35]. Nevertheless, it can be inferred that the occurrence of ALDH^high^ cells is underrated in tissues in which the chief ALDH isozyme is not a member of the ALDH1 family.

Studies on a variety of cancers such as prostate cancer, ovarian cancer, breast cancer, lung cancer-derived primary culture, and nonmetastatic/metastatic cell lines of these cancers suggest variation in ALDH expression [36–47]. This inconsistency could refute potentially applicable CSC markers and also brings into limelight the significance of testing in multiple cell lines/primary tissues. The clinical outcome in a variety of cancers has been attributed to ALDH expression [48–50]. ALDH has been implicated in resistance mechanisms to radiotherapy and chemotherapy; thus, a more specific, diverse therapeutic approach must be embarked upon. Some research groups have been attracted towards investigating inhibitors and involved in developing more specific inhibitors through drug discovery approaches, as the common expression of ALDH in healthy stem cells which can cause an off-target activity [35]. It is possible that functional redundancy within the isozyme family could recompense the inhibition for one ALDH. ALDH^high^ CSC-loaded dendritic cells (DCs) have been used fruitfully in two in vivo melanoma models in the immunotherapy setting [51, 52]. In transplantation setting, ALDH activity has been demonstrated in the T regulatory cell (*T*_reg_) immune subset; thus, targeting of ALDH may also show an antitumor impact through modulation of *T*_reg_ subset [53].

CSCs possess a lesser number of mitochondria and consequently show more glycolytic activity than other tumor cells, as evident in liver, breast, melanoma, and lung cancer [54–57]. It can be perceived by attenuated mitochondrial activity, diminished intracellular concentrations of reactive oxygen species (ROS) and ATP, perinuclear mitochondrial distribution, and a smaller concentration of mitochondrial DNA in CSCs [58]. CSC inhibitors have promising off-target effects on other cell types focussing on either self-renewal pathways, surface markers, or enzymes.  Table 2 illustrates the potential off-target activities of some of these inhibitors on immune cells [59–61].

### 3.3. Identification on the Basis of Functional Alteration

CSCs can be functionally distinguished from SCs by the fact that they exhibit a sluggish rate of cell division, amplified drug, and radiotherapy resistance and display an activation of detoxification pathways which forms the basis for their identification as well.

Characteristic staining of retaining dyes like PKH, carboxyfluorescein succinimidyl ester (CFSE), or bromodeoxyuridine (BrdU) that mostly become dilute during the proliferation phase of the cell cycle can be delineated by the poor rate of cell division of CSCs especially in growth preparatory phase or G0. These dye retaining cells give rise to xenotransplants in a number of cancers of the breast, melanoma, pancreas, and glioma [62–66].

The incremented intensity of drug resistance has been found in CSCs due to detoxifying pathways. ABCB1, ABCB5, ABCG2, and ABCC1 which are the members of ATP binding cassette transporter family of proteins are active in CSCs and inactive during differentiation [67]. They function to pump out complex molecules from the cell cytoplasm, thereby, shielding the cells from exogenous toxins like various drugs utilized for chemotherapy. Peptides, lipids, proteins, polysaccharides, and a number of diverse hydrophobic drugs act as their substrates [68]. Targeting them with highly selective and specific inhibitor molecules remains a research niche that attracts the interest of cancer researchers all over the world [69]. Hydrophobic Hoechst dyes are also excluded/expelled by CSCs owing to this mechanism; a side population based on low dye levels is formed by CSCs which aids in their identification [70]. Certain ABCB proteins such as transporter associated with antigen processing (TAP protein) play an instrumental part in intracellular trafficking of peptides across the membranes with major association with dendritic cells (DCs) and major histocompatibility complex (MHC) class I function [71]. As a consequence, the off-target effect of tumor ABCB targeting may be lethal for generating proficient antitumor T-cell responses.

There is strong experimental and clinical evidence that CSCs are intimately involved in both intrinsic and acquired tumor resistance to anticancer treatments including radiotherapy. Unraveling the underpinning mechanisms that govern the maintenance of CSCs and their resistance to therapy continues to remain a daunting task. Radiation damages the DNA by causing single- or double-strand breaks or opting an indirect route through ROS and may selectively kill the relatively radiosensitive tumor cell populations leaving the therapy-resistant CSCs alive, thus leading to temporary or permanent cell cycle arrest via the selective repopulation from the surviving CSCs through DNA damage repair (DDR). DDR arbitrates senescence or cell death if the damage is irreparable [72]. The cell may die if the antioxidant defence mechanism is not able to bring out the cell from the state of oxidative stress caused by ROS. Intrinsic radioresistance in CSCs can be majorly attributed to both the two highly efficient pathways, namely, DDR and ROS operating in them. Extrinsic or acquired radioresistance may be ascribed to the localization of CSCs in hypoxic zones inside the tumor. Survival of CSCs is fostered to hypoxia-inducible factors (HIF) 1*α* and HIF2*α* which have been shown to activate Sonic Hedgehog, Wnt, and Notch pathways for stem cell renewal and multipotency. Also, an accumulating body of evidence demonstrates that CSC rich cancers respond negatively or less efficaciously to radiotherapy [73, 74]. Nevertheless, the impact of hypoxia is not inhibitory on immune cell function. HIF1*α* regulates long-term survival of activated DC. HIF2*α* turns on the cytolytic machinery of effector T cells that are hypoxia resistant. Effector T cells displaying glycolytic characteristics exert their effector function even in the areas with poor vasculature, may be where CSCs reside. Low-dose radiations harm naïve T cells unlike the memory T cells and effector T cells [75–77]. Blending radiotherapy with T-cell targeting of stem cells can be more fruitful stratagem which would impart promising synergistic effects.

CSCs may, however, be abolished by high-dose radiations as witnessed in prostate and lung cancers of the early stage. Partly, it may occur due to immunogenic cell death, which commences by uptake of tumor antigens and antigen cross-presentation by DC [78]. It can be contemplated if CSC death due to radiation is immunogenic or if radiation gives rise to specific effector and memory T cells leading to abscopal effect and ultimate failure to recur. Lots of scientific minds are engrossed to address this predicament of paramount clinical significance.

## 4. Immunomodulating and Immunoresistance Properties of CSCs

### 4.1. Immunomodulation

CSCs show immunomodulation mainly through immunosuppression of immune cells. *In vitro* bioassays displayed an exemplary instance for the inhibition of proliferative T-cell responses and IL-2 production by CSCs in melanoma and glioblastoma. Treg frequencies were not increased in glioblastoma and increased in melanoma CSC-T-cell cocultures [79, 80]. Immunosuppression of antigen-presenting cells, Natural Killer (NK) cells, and T cells is exerted by tumorigenic growth factor- (TGF-) *β*, Interleukin- (IL-) 4, IL-13, and IL-10 secreted by CSCs [80–82].

Immune responses are weakened by cell surface molecules on CSCs like CD200. Experiments on breast cancer *in vivo* model have revealed that minimised T helper (Th)1 responses, elevated IL10 production, and attenuated neutrophil infiltration are credited to CD200 overexpression [83, 84]. Overexpression of programmed death ligand 1 (PD-L1) in cancers is associated with increased glycolytic behaviour. The high expression on CSCs triggers PD-L1 upregulation that is plausibly localization dependent or tumor type [85]. Melanomas do not express high eminent levels of PD-L1 but overexpression is a conspicuous feature in gastric, head, and neck and CD133^+^ colorectal cancers [61, 79, 86, 87].

### 4.2. Immunoresistance

In contrast to non-CSCs, glioblastoma CSCs display downregulation of MHC class I. On the other hand, autologous T-cell responses were induced *in vitro* by these CSCs. Moreover, the vulnerability of CSCs to T-cell-mediated immune responses is boosted by IFN-*γ*-treatment [80]. Even though the feasibility of therapeutic vaccines in clinical settings lies in infancy, cancer vaccines that generate T-cell and antibody responses against tumor-associated antigens (TAAs) yield promising inferences in preclinical models. Vaccine specificity for CSCs antigens is majorly lacking which could also contribute to increased resistance to T-cell attack. Development of stem-like features like Nanog in surviving cancer cells subsequently after vaccination has been reported [88]. Silencing Nanog could aid in getting rid of T-cell resistance. This sheds light on the fact that stem-like features may build up in a cell population as a consequence of immunological stress; however, it does not show inherent SC resistance.

The evidence that purified CSCs are employed as vaccines brings an end to the entire dispute that cancer stem-like cells may not be inherently resistant to immune attack. T cells generated through this treatment provide better protection in the D5 melanoma model against pulmonary metastasis in compression to the vaccination using unseparated cells. This study along with others proves T-cell susceptibility and indicates the feasibility of CSCs targeting by T cells [51, 89].

## 5. Immunological Targeting of CSCs

Immunotherapy targeting tactics chiefly focus to hamper the immunosuppressive TME, disrupt immune-repressive regulatory networks, and activate cytolytic lymphocytes [90]. A gamut of strategies targeting immune responses in cancer cells and CSCs, in general, has been investigated. However, such immunotherapy aiming strategies in CSCs are in the budding phase in the preventive preclinical stage and need to be investigated further so that they can be taken from bench to bedside. Here, we are discussing some possible strategies to immunologically target CSCs.

### 5.1. Innate Immune Response to CSCs

An appropriate candidate for immunotherapy of hematologic and solid types of tumors is NK cells that are major effector cells for innate immunity [91, 92]. Nonetheless, the exact participation of NK cells in anti-CSC immune surveillance remains discordant. Wu and his coworkers scrutinized the immunogenicity of CD133^+^ brain tumor stem cells (BTSCs) and showed that MHC I or NK cell-activating ligands fail to be expressed by a large proportion of CD133^+^ cells, thereby making them defiant to innate and adaptive immune surveillance [93]. Wang et al. verified that the immune CSCs in breast cancer evade NK cell killing owing to the marginal expression of MICA and MICB (MHC class I-related chains A and B), two ligands for the stimulatory NK cell receptor NKG2D due to aberrant expression of oncogenic micro-RNA miR-20a [94]. On the other hand, a research group led by Castriconi accounted that a range of ligands of NK cell activation receptors are expressed by glioma stem cells (GSCs) which evokes optimal NK cell cytotoxicity. They deduced that GSCs fall prey to lysis mediated by both allogeneic and autologous IL-2 (or IL-15)-activated NK cells [95].

In another investigation by Tseng et al., it was inferred that NK cell-mediated cytotoxicity may harm primary oral squamous carcinoma stem cells (OSCSCs) more significantly as compared to their differentiated counterpart [96]. Similarly, allogenic NK cell recognition of colorectal adenocarcinoma CSCs was analyzed by Tallerico et al. and also showed that these CSCs are more predisposed to NK cells in contrast to their non-CSC counterpart. Elevated levels of ligands for natural cytotoxicity receptors in colorectal CSCs make them more vulnerable to NK cell killing as compared to non-CSCs [97].

Exceptional *γδ* T cells comprise 1–5% of circulating lymphocytes and are of V*γ*9V*δ*2 phenotype and are part of innate effector group family. They are characterized by potent non-HLA restricted cytotoxicity against tumor units and identify and present antigens to *αβ* T cells; hence, immunotherapy targeting *γδ* T cells is grabbing the attention of the scientists worldwide [98, 99]. *γδ* T cells target isopentenyl pyrophosphate (IPP) which is crucial for isoprenoid biosynthesis in eukaryotes [100, 101]. Cytolysis of sphere forming neuroblastoma cells sensitized to zoledronate is mediated by V*γ*9V*δ*2 T cells as demonstrated by Nishio and his group [102]. Similarly, zoledronate-sensitized human colon CSCs cause V*γ*9V*δ*2 T cells to exhibit an increased rate of proliferation, tend to secrete TNF-*α* and IFN-*γ*, and produce the apoptotic and cytotoxic molecules granzymes and TRAIL [103]. Increased CD69 expression by activated V*γ*9V*δ*2 T cells after zoledronate exposure was shown in a clinical study. Decreased expression of the lymphoid-homing receptors CCR7 and CXCR5 was observed in V*γ*9V*δ*2 T cells. On the contrary, upregulated peripheral tissue-homing chemokine receptors CCR5 and CXCR3 were also displayed by V*γ*9V*δ*2 T cells [102]. *In vitro *these V*γ*9V*δ*2 T cells were found cytotoxic. These transferred V*γ*9V*δ*2 T cells homed predominantly to the spleen, lung, and liver and to the metastatic sites outside these organs [104]. This signifies that *in vitro* expansion of the autologous *γδ*T cells together with different antineoplastic drugs may be for the betterment of cancer treatment via CSC obliteration. Further investigations to substantiate direct targeting of CSCs by *γδ* T cells are warranted. The dearth of clinical data relating to the use of nonspecific killer cells in the adoptive immunotherapy of cancers mediated by CSCs is the major lacuna in this area and needs to be addressed at the earliest.

### 5.2. Antigen-Specific Targeting by T-Cell Immunotherapy

The T-cell based immunotherapy needs to generate effector T cells followed by adoptive transfer of CD8^+^ T cells back into patients. Here, we are describing the routes to generate CSC-specific T cells that include CSC-primed T cells and genetic engineering of T cells with chimeric antigen receptors (CARs).

Regarding CSC-primed T cells, CD8^+^ T-cell response could be elicited by employing CSCs derived from breast, pancreatic, and head and neck cell lines [105]. For instance, an *in vitro* stimulus of human CD8^+^ T cells secluded from peripheral blood of normal HLA-A2^+^ donors with ALDH1A1 peptide-pulsed autologous DCs could trigger ALDH-specific CD8^+^ T cells. Remarkably, animal survival after adoptive transfer in preclinical bioassays can be extended by these ALDH-specific CD8^+^ T cells that can identify and abolish ALDH^hi^ CSCs (decreased by 60%–89%) *in vitro* and restrain metastasis and xenograft growth *in vivo* [105].

Likewise, lung CSCs were isolated as ALDH^hi^ population and exploited for lysate-pulsed DCs to stimulate CD8^+^ T cells by cocultivation as demonstrated by Luo et al. [106]. Subsequently, noteworthy anticancer effects, ensuing in retarded tumor growth and increased survival, were revealed by these ALDH^high^-CD8^+^ T cells [106]. Moreover, HLA-I molecules and autologous cytotoxic T lymphocytes (CTLs) were expressed by CSCs that were purified as SP from bone malignant fibrous histiocytoma (MFH) *in vitro* coculture with SP cells [107]. Also, an important indication that the CTL clone specifically recognizes MFH CSC-specific antigens came from the observation that CTL clones are biased towards the recognition of SP cells rather than non-SP cells [107].

Nevertheless, it is imperative to note that CSC-primed T cells distinguished antigens are predominantly unidentified. Otherwise, CAR T cells symbolize a unique and a potential cancer immunotherapy. The past few decades have witnessed a furious attention of the scientific community towards applying this therapy in solid tumors which is driven by the profound success and advances in the treatment of hematological malignancies using the adoptive transfer of CAR T cells. T cells can be genetically crafted employing ex vivo gene transfer for specifically recognizing a TAA or for expressing the novel T-cell receptor, hence, arbitrating neoplastic cells. Consequently, CAR T cells can be engineered to target virtually any TAAs to recognize cell surface protein in an MHC-unrestricted manner [108].

In the preclinical experimental setting of solid tumors, CAR T cells have been perceived to target CSC-associated antigens, such as CSPG4 in many discrete types, EpCAM in prostatic carcinoma (PCa), CD133 in glioblastoma, and EGFRvIII and IL13R*α*2 in gliomas [109–113]. Antitumor impacts of CAR T cells mediated by CSCs are highlighted through these studies although there is a paucity of studies in this area. Significantly, an elevated rate of toxicities has been observed for CAR *T* therapies targeting TAAs including ERBB2 in metastatic colon cancer and carbonic anhydrase IX (CAIX) in renal cell carcinoma apparently accredited to the common expression of targeted antigens by both cancer and normal cells [114, 115]. Isolation and expansion of T cells restricted to specific TAAs are a daunting task due to technical cumbersomeness. A meticulous approach towards recognition of CSC-specific antigens is expected to be vital in this approach for targeting CSCs. Propitious candidacy for focussing on CAR *T* therapy is of neoantigens that can be acknowledged skillfully by T cells without self-tolerance mechanisms.

Although the existing knowledge on CSC genomics is in its nascent phase, studies suggest the use of *in vitro* generated and expanded CSC-specific T cells for adoptive transfer into tumor-bearing hosts *in vivo* to target CSCs for eliminating the tumor. Theoretically, *in vitro* CSC-primed T cells may represent a newer and pragmatic immunotherapy to precisely focus on CSC [116] but practically this approach is still under the developmental stage.

### 5.3. CSCs Vaccines

The immunotherapeutic strategies are based on the activation of T-cell responses endogenously to target malignant cells via transferring TAAs to patients, which can be successfully achieved by vaccines such as whole cell, genetic, DC, and peptide. At present, the most efficacious and most interrogated strategy to prevent diseases is DC vaccination. Ex vivo induction of DCs, the professional antigen-presenting cells (APCs) from peripheral blood monocytes or marrow cells, pulsed with tumor antigens, maturated, and eventually administered to the patient. Comprehensive data from different experiments have thrown light on the use of DCs to initiate tumor-specific T-cell responses which epitomises a highly potential cancer vaccination approach [116–118]. In particular, DC-mediated tumor-specific immune responses have been fostered by CSCs that function as antigen sources [119, 120]. In principle, the employment of CSC lysates as an antigen source could facilitate synchronized targeting of diverse antigens and thereby be less prone to antigen loss due to tumor escape [116]. Also, non-CSCs are less effective in inducing antitumor immunity against CSC epitopes as compared to enriched CSCs that are immunogenic and better as an antigen source. For example, the fact that can be considered is that, in various syngeneic tumor models of immunocompetent mice, the CSC-DC vaccination notably circumvents lung metastasis and slows down the growth of squamous carcinoma compared to immunization with bulk tumor cells [51]. The vaccine comprised of DCs pulsed with irradiated PCSC (prostate cancer stem cells) lines derived from the TRAMP (transgenic adenocarcinoma of the mouse prostate) tumors induces a more robust tumor-specific immune response than the response induced by DCs pulsed with differentiated prostate tumor cells. It has also been observed that the CSC-DC vaccine retards tumor growth [121].

In a study, cytotoxic T lymphocytes (CTL) antitumor response and activation of CD8^+^ and CD45^+^ T cells were also credited to the breast CSC-DC vaccine [122]. Fascinatingly, it has been observed that, in the postsurgery phase, CSC-DC vaccines are extremely efficacious when deployed in an adjuvant setting to eradicate microscopic CSCs, or as combinatorial therapy with radiation and/or chemotherapy in treating macroscopic tumors. This was observed in established murine melanoma D5 and squamous cancer SCC7 tumor models where CSC-DC was more efficient when compared to DCs pulsed with non-CSCs in curing microscopic tumors in [123]. Furthermore, ALDH^high^ CSC-DC vaccines in the adjuvant setting were more promising than traditional DC vaccines as they reduced tumor relapse and lung metastasis coupled with enhanced host survival further and obstructing PD-L1 [124]. DNA vaccination is gaining hype as compared to conventional cell-based vaccines, as they involve injecting plasmids for direct antigen production culminating in a protective immunological response, thus preventing cancer [125]. Acceptability of DNA immunization and their aptitude to skilfully trigger antigen-specific T cells has been efficiently established by clinical trials. PAP and PSA have been acclaimed as DNA-based vaccines in human castration-resistant prostate cancer (CRPC) [126, 127]. In a study, Nishizawa et al. observed that, in comparison to immunization with the TAA survivin, immunization with CSC-specific DNAJB8 expression plasmids shows better antitumor immune response [128].

Recently, an experimental DNA vaccine has been developed against sternness-specific marker Sox2 and an antitumor effect also has been seen after immunization against murine lung TC-1/B7 cancer cells expressing oncogenic Sox2 [129]. In a nutshell, it can be contemplated that given the ease and theoretical applicability to target antigens, CSC-specific DNA vaccination holds promise to serve as a tool for immunotherapy.

### 5.4. Antibody-Based Immunotherapy

The past two decades have brought into limelight the significance and feasibility of monoclonal antibodies- (mAb-) based treatments as therapies against cancer treatment. Antibody-drug conjugates are powerful newer discovered weapons for the fight against cancer [130], and immunomodulatory antibodies, illustrated by anti-PD-L1, anti-PD-1, and immune checkpoints targeting anti-CTLA-4 antibodies, have also recently attained outstanding clinical success (discussed in immune checkpoints). The expression levels of some markers (Table 1) in CSCs considerably dissimilar to the other tumor cells, which provides promising targets for antibody-based immunotherapy. The endeavours targeting CSCs with specific antibodies have been highly fruitful in terms of therapy response. For instance, tumor progression is decelerated and apoptosis is triggered in leukemic stem cells by an anti-CD44 mAb [131, 132]. Similarly, human melanoma metastasis is lessened, animal survival in SCID mouse is enhanced, tumor growth reduced, and apoptosis commenced in murine breast tumors due to anti-CD44 antibodies [133, 134].

Certainly, targeting CSCs with antibodies has proved to be beneficial in improving treatment responses. Also *in vivo* tumor growth and *in vitro* proliferation of CD133^+^ gastric and hepatocellular CSCs can be subdued by drug-conjugated anti-CD133 antibody [135]. A bispecific antibody consisting of CD133 and CD3 antibodies which were asymmetric in nature displayed a strong antitumor efficacy in a variety of tumors [136, 137]. Studies have revealed that antitumor capability in solid tumors can be made better with a CSC-specific antibody-incorporated liposomal nanoparticle delivery system loaded with drugs or a suicide gene [138, 139]. In yet another classic example, trastuzumab (HER2-targeting antibody) dramatically lessens the chances of breast cancer recurrence by targeting HER2, an important regulator of breast CSC self-renewal [140]. Similarly, other HER2-targeting agents, like monoclonal antibody pertuzumab and the immunotoxin conjugate ado-trastuzumab emtansine (TDM-1), have further upgraded the efficacy of human epidermal growth factor receptor (HER2) targeting in the clinical settings [141, 142].

Outstandingly, HER2 expression can also be targeted in GBM CSCs [143]. As the “CSC markers” may not necessarily be unique to CSCs, single-agent mAb therapy may adversely affect normal cells [130]. Combination therapy using an assortment of different antibodies targeting multiple CSC markers could potentially reduce doses of individual antibodies to accomplish the efficient abolishment of CSCs while reducing side effects due to huge concentrations of single anti-CSC mAbs.

### 5.5. Tumor Microenvironment Targeting Immunotherapy

There are few important factors present in the surrounding tumor microenvironment of CSCs such as hypoxia, chronic inflammation, inflammatory cytokines, and perivascular niches (role in the regulation of proliferation and differentiation) [144–146]. Stat3/NF-*κ*B pathways in tumor and stromal cells are activated by inflammatory cytokines such as IL-1*β*, IL-6, and IL-8 to further secrete cytokines in a positive feedback loop that elicits angiogenesis, CSC self-renewal, and metastasis [144, 147]. In addition, the CSC population which coevolved in the tumor microenvironment are adjacent blood vasculature that forms a niche defined by severe enhanced angiogenesis and hypoxia [145, 146]. These facets of the tumor microenvironment have been deciphered as probable pharmaceutical targets of CSCs.

Recent studies have verified that reduced tumor growth is achieved via IL-6 and/or IL-8 cytokine signalling blockade [148, 149]. Repertaxin, a pharmaceutical commodity, a noncompetitive inhibitor of IL-8 and CXCR1 signalling, could decrease tumor size and enhance chemotherapy efficacy in breast cancer model [150]. However, it has been seen that blocking single cytokines induces limited effects as both IL-6 and IL-8 are crucial for tumor growth and the expression of these genes combined with dismal prognosis in breast cancer patients. Hence, simultaneously inhibiting IL-6 and IL-8 expression was a more lucrative line of action to induce appreciable changes in tumor growth [151].

Tumor hypoxia is another fascinating means to hit the CSC niches. Hypoxia causes chemo- and radioresistance by activating the HIF pathway and upregulating HIF-1*α*, by mediating multiple biological effects of hypoxia in tissues. Albeit HIF pathway inhibitor molecules have been underway in clinical trials, they seldom become successful enough to cross the clinical trial stage [152]. Also, the CSC niche can be dislocated by targeting tumor vasculature. Limited success has been achieved from the clinical trials targeting angiogenesis by blocking the vascular endothelial growth factor pathway [145].

### 5.6. Immune Checkpoints

Immune checkpoints are known for declining autoimmunity by intervening coinhibitory signalling pathways [153]. In cancer, these inhibitory pathways are believed to be implicated in tumor immune resistance [154]. PD-1/PD-L1 axis and the cytotoxic T lymphocyte antigen 4 (CTLA-4)/B7 axis are the two instrumental pathways of immunoinhibition that have been identified to date. These negative mechanisms contribute to the development of a suppressive microenvironment rendering cell resistance to immune therapy [155, 156]. CSCs might secrete paracrine factors or direct cell-cell contact and jointly transform the immune cells in the CSC niche because physiologic stem cells have immunoprivilege and active immunoregulatory functions [3, 157–160]. Schatton et al. accounted that T-cell activation is downregulated by CSCs [79, 161]. They identified malignant melanoma initiating cells (MMICs), a novel type of CSCs, on the basis of the expression of ABCB5 (chemoresistance determinant) [161]. The human ABCB5^+^ MMICs express PD-1 and B7.2 and low expression of PD-L1 in comparison to ABCD5^–^ cells. The clinical benefit of PD1 and PD-L1 was witnessed in Hodgkin's disease, melanoma, and lung cancers [162–164]. A better-prolonged response was observed in a patient subset that was considerably more durable compared to targeted or cytotoxic therapies.

It is proposed that activated T-cell responses may be downregulated by tumor expression of PD-L1 through the PD-L1/PD-1 axis and its blocking results. Even though Schatton et al. suggested reduced expression of PD-L1 in MMICs [79], in another study, a better expression of PD-L1 was observed in CSCs of head and neck carcinoma [165]. It can be inferred from this that CSC subsets may reduce T-cell response through the PD-1/PD-L1 axis. However, future clinical trials on immune checkpoint blockade are mandatory for the establishment of these results. Also, the synergistic effects of immune checkpoint therapies with CSC-targeting immunotherapies are suspected to magnify the clinical applicability of each approach.

## 6. Limitation and Challenges

It has been found that owing to stemlike features like low immunogenicity and intrinsic conventional therapy resistance, CSCs are demonstrated to be participants in the processes of tumor progression, maintenance, metastasis, and relapse. Consequently, CSCs form a crucial target to treat residual disease and circumvent the process of recurrence. Immunotherapy is a pragmatic promising tool as evidenced by the results of a number of clinical trials on cancer patients. However, in the clinical trials, it has been shown that the objective response rate varies significantly and a durable response is often confined to a small patient population. It is important to note that the results of the current mono-immunotherapies in solid malignancies are generally unsatisfactory. One of the fundamental reasons for this inadequacy might be due to the presence of CSCs that are not efficiently targeted by the available regimens for immunotherapy. The concept of CSC-specific immunotherapy remains in its budding phase, although it has established utility in a few preclinical and clinical trials (Table 3); however, challenges still exist. In order to fabricate more promising strategies and novel therapeutics, genomic, immunological, and biological characterization of CSCs along with immune cell interactions in the TME is warranted. Despite the fact that considerable knowledge has been accumulated on human CSC properties, most of our current understanding is attributed to derived from xenograft studies in immune-compromised nude mice. In the future, employing humanized mice, immunodeficient strains with engrafted human immune systems, may help to throw light in this area [166]. Yet, another hurdle is the heterogeneity in the population of CSCs and the property of plasticity of cancerous cells. CSCs are acknowledged as heterogeneous [2] revealed by the fact that different subpopulations of CSCs sometimes express different phenotypic markers in a single cancer type. For example, a previous research group has confirmed that prostate CSCs are mainly PSA‐/lo but this is rather heterogeneous inhabiting a vast array of tumorigenic cell subsets that can be prospectively purified out using distinctive markers [167].

Data amassed from different laboratories worldwide ascertains that the association between various CSC subsets within a similar cancer type remains largely unidentified, and if CSC subpopulations share common immunological features is also an area to be explored. Hence, there may be a possibility of a situation that a CSC-specific immunological treatment leads to the eradication of a particular subset of CSCs not all. Furthermore, CSCs and non-CSCs can be thought to exhibit diverse plasticity (not discussed in this review but reviewed in [2]). This tumor cell plasticity is a major impediment in the establishment of long-lasting and more effective targeted cancer therapies, because therapeutic annihilation of present CSC populations might be then followed by their regeneration from non-CSC origin within the tumor due to treatment pressures [2].

It is gripping to see that various agents aim at targeting TME components that demonstrate clinical relevance in the treatment of cancers, as our present knowledge about the TME is limited. Notably, current research studies have advocated that conventional therapies of cancer are more prone to augment CSCs and restructure the TME which may modify the immunotherapy responding ability of CSCs [3]. For instance, chemotherapy enhanced the frequency of the CSCs in the tumors and downregulated the expression of HLA1 molecules in HNSCC and PCa [168, 169] that may cause immunoresistance. Collectively, a CSC epitomises a continuously reshaping target, as it keeps on developing along with tumor development and progression, particularly, under the influence of treatment. It can be concluded that the area of immune targeting of CSCs holds noteworthy promise in curing patients with cancer. While therapies that efficiently and selectively get rid of CSCs do not have clinical applicability, researches on various immunotherapeutic strategies to target CSCs (Table 3) are in progress and many of them have displayed efficacy in diminishing tumor growth and metastasis in preclinical and clinical settings.

Like all the monotherapies, mono-immunotherapy is improbable to treat cancer, and stratagem that merges both conventional therapies and CSC-specific immunotherapies would be attractive and promising tactics to combat the deadly disease cancer. Combinatorial approaches can reduce drug resistance and cancer cell plasticity and help attain efficacious treatment outcomes as compared to the monotherapies. Theoretically speaking, the immunogenicity of CSCs can be incremented by the inhibition of negative immunoregulatory pathways and through the upregulation of APM and HLA-I components via a combination of treatment strategies with radiotherapy, chemotherapy, IFNs, and/epigenetic therapies [8]. For example, CAR T cells specific to CSCs along with other therapies could be effective in increasing their antitumor effect [170]. It has been reported that epigenetic drugs alter the expression levels of genes related to the immune system on either tumor cells and/or tumor-associated immune system cells in a way that replenishes the immunogenicity and immune recognition capability. For example, APC functions improved T-cell activation promoted by HDAC6 inhibitor ricolinostat in NSCLC cells, as a result of modulation of expression of MHC molecules, while CD4+FOXP3+ Treg cell suppressive functions are pacified and immune-mediated tumor growth arrest is facilitated and these are attributed to JQ1, a BET bromodomain inhibitor [171].

Henceforth, epigenetic therapy in combination with immunotherapy may represent a novel paradigm in cancer care and therapeutic intervention. In the coming future, meticulous assessment of these strategies either alone or coalescing with myriad treatments is required to shed light on the establishment of novel antineoplastic immunotherapy treatment regimes.

## 7. Conclusion

Owing to their intrinsic stemness that renders CSCs therapy resistant and repeatedly immunocompromised, they are believed to be vitally involved in tumor maintenance, progression, recurrence, and metastasis. Hence, targeting CSCs is indispensable for treating residual disease and for circumventing relapse. The results of clinical trials registered on http://clinicaltrials.gov website which are using immunotherapy to target CSCs are not yet available; therefore, it can be said that various immunotherapeutic strategies to target CSCs are at the developmental stage. These approaches include targeting of CSCs with immunological methods such as CSC-DC vaccine, targeting the tumor microenvironment, anti-IL-6 mAb, inhibition of CSC-mediated immune-suppression, and blocking through anti-PD-1/PD-L1 mAbs. As an obligation and a mandatory step, rigorous tests need to be undertaken to check these strategies either alone or in combination to further verify their therapeutic worth. However, immunologic targeting of CSCs symbolizes a novel potential approach in cancer therapeutics which we hypothesize will be more efficient in amalgamation with conventional modalities and agents having immunomodulatory property.

## Figures and Tables

**Table 1:** CSC Markers in different cancers.

Cancer type	CSC markers reviewed in [1, 3, 6–8]	
	Positive markers	Negative markers
Leukemia	CD34, CD96, ALDH, CD47, CD44, CD123, TIM-3, CD32, CD25, CLL-1	CD38
Prostate	ABCG2, ALDH, CD44, *α*2*β*1, CXCR4, CD133, NANOG, TRA-1-60, CD151, CD 166	PSA, CK18,
Bladder	CD44, CK5, CD44v6, 67LR, CK17, ALDH, SOX2	CK20, EMA, CD66c,
Breast	CD44, PKH26, CD49f, ALDH, CD133, CD90	CD24
Lung	CD133, ALDH, CD117/c-kit, OCT4, NANOG, CD44, TPBG/5 T4, CD166, CD44, EpCAM	
Pancreatic	CD44v6, *α*6*β*4, TSPAN8, CXCR4, CD44, CD24, ESA, c-Met, ALDH, CD133,	
Glioblastoma	CD133, ABCG2, SSEA-1, SOX2, BMI1, MUSASHI1, NESTIN, OLIG2, CD49f, A2B5, L1CAM, EGFR, CD44, ID1, MYC, ALDH	
Ovarian	ALDH, CD44, CD117/c-kit, CD44, MyD88, CD24, CD133, CXCR4	
Colon	CD133, EpCAM, CD44, CD166, ALDH, LGR5, ABCB5	
Liver	CD133, ALDH, EpCAM, CD90, CD44, CD13, SALL4	CD45

**Table 2:** Off-target effect of CSC-targeting inhibitors on immune cells.

Inhibition of CSC pathways, function and markers	Off targeting effect on immune cells	Reference
*β*-catenin	Impairs polarization, differentiation, and maturation of T cells	[23, 24]
Notch receptor	Blocks cytotoxic T cells' function and cytotoxin production	[19, 20]
WnT receptor	Inhibits CD8 positive T cell's development	[23, 24]
Hedgehog receptor	Inhibits myeloid-derived suppressor cell function	[30]
ABCB complex	Inhibits transporter associated with antigen processing (TAP) in antigen-presenting cells	[69]
ALDH	Inhibits regulatory T cells' function	[52]
IL-6 receptor	Impairs central and naïve memory T cells' proliferation, survival, and effector function	[59]
CD44	Impairs Th1 cell survival, memory, effector function, and IFN*γ* production	[60, 61]

**Table 3:** CSC-targeting strategies and their effect in different types of cancer.

Type	Cancer types	Effects of the CSC-targeting strategies	Reference
CSC-primed T cells	Head and neck	ALDH1A1-specific CD8^+^ T cells distinguish and eradicate ALDHhi CSCs in *in vitro* bioassays, retard xenograft growth and metastases in *in vivo* bioassays, and prolong survival	[105]
	Lung	ALDH^high^-CD8^+^ T cells resulted in the inhibition of tumor growth and prolonged survival, hence, bestowing more considerable antitumor effects	[106]

CSC-lysate DC vaccine	Squamous cell Carcinoma/Melanoma	CSC-DC vaccine that was administered in the adjuvant setting after localized radiation therapy of established tumors resulted in a reduction of tumor growth, and vaccination significantly inhibited tumor growth, abridged ALDH^high^ CSC frequency in primary tumors, and metastases through stimulation of humoral immune responses against CSCs	[123]
		SCC growth was regressed compared to immunization with bulk tumor cells and lung metastasis of melanoma cells was appreciably curtailed	[51]
		In the adjuvant setting, simultaneous PD-L1 blockade further enhanced local tumor recurrence and spontaneous pulmonary metastasis and also increased survival of the host	[125]
	Prostate	Tumor regression was witnessed in TRAMP mice, tumor growth was delayed in mice challenged with prostate CSCs, and tumor-specific immune response was induced that was stronger than differentiated tumor cells	[121]
	Glioblastomas	Antigen-specific T-cell responses against CSCs were elicited and survival in animals was improved	[120]
	Breast	Migration of DCs to the spleen activated CD8^+^ and CD45^+^ T cells; in turn, CTL antitumor responses were induced	[122]

CSC-mRNA-DC vaccine	Glioblastomas	Seven patients vaccinated with an mRNA-DC vaccine exhibited a common immune response	[172]

DNA vaccine	Renal cell carcinoma	Stronger antitumor effects were observed in immunization with DNAJB8 expression plasmids in contrast with immunization with the tumor-associated antigen survivin, which was expressed in both CSCs and non-CSCs	[128]

NK cells	Glioblastomas	Neural stem cells derived from tumor specimens were prone to attack by lysis mediated by both autologous IL-2 (or IL-15) activated NK cells but resisted freshly isolated NK cells	[95]
	Pancreatic/Breast/Glioblastomas	CSCs isolated from an array of human cancer cell lines *in vitro* and dissociated primary cancer specimens ex vivo were preferentially targeted by allogenic activated human NK cells	[173]

mAb	Liver/Pancreatic	The growth of hepatic and pancreatic cancer cells was inhibited *in vitro* and *in vivo* and CD133 high CSCs were targeted by CIK cells bound with anti-CD133/anti-CD3 bispecific antibodies	[137]
	Melanoma	Human melanoma metastasis was inhibited and the survival of tumor-bearing animals was prolonged by anti-CD44 antibodies	[133]
	Breast	Murine breast tumor growth was inhibited and apoptosis was induced by anti-CD44 antibodies	[134]

CSC-CAR T	Glioblastomas	Patient-derived GBM CSCs were annihilated in an orthotopic tumor model and *in vitro* by anti-CD133 CAR T cells	[112]
	Prostate	Significant antitumor efficacy was exhibited by EpCAM-specific CAR T cells *in vitro* and *in vivo* systems	[113]
